# ER-driven membrane contact sites: Evolutionary conserved machineries for stress response and autophagy regulation?

**DOI:** 10.1080/19420889.2017.1401699

**Published:** 2017-12-13

**Authors:** Diana Molino, Anna Chiara Nascimbeni, Francesca Giordano, Patrice Codogno, Etienne Morel

**Affiliations:** a Institut Necker-Enfants Malades (INEM), INSERM U1151-CNRS UMR; b Université Paris Descartes-Sorbonne Paris Cité, Paris, France; c Institute for Integrative Biology of the Cell (I2BC), CEA, CNRS, Univ. Paris-Sud, Université Paris-Saclay, Gif-sur-Yvette, France

**Keywords:** autophagosome, contact-sites, ER membrane dynamics, mitochondria, plasma membrane

## Abstract

Endoplasmic Reticulum (ER), spreading in the whole cell cytoplasm, is a central player in eukaryotic cell homeostasis, from plants to mammals. Beside crucial functions, such as membrane lipids and proteins synthesis and outward transport, the ER is able to connect to virtually every endomembrane compartment by specific tethering molecular machineries, which enables the establishment of membrane-membrane contact sites. ER-mitochondria contact sites have been shown to be involved in autophagosome biogenesis, the main organelle of the autophagy degradation pathway. More recently we demonstrated that also ER-plasma membrane contact sites are sites for autophagosomes assembly, suggesting that more generally ER-organelles contacts are involved in autophagy and organelle biogenesis. Here we aim to discuss the functioning of ER-driven contact sites in mammals and plants and more in particular emphasize on their recently highlighted function in autophagy to finally conclude on some key questions that may be useful for further research in the field.

## ER membrane dynamics and associated contact sites in autophagy

**Figure 1.**
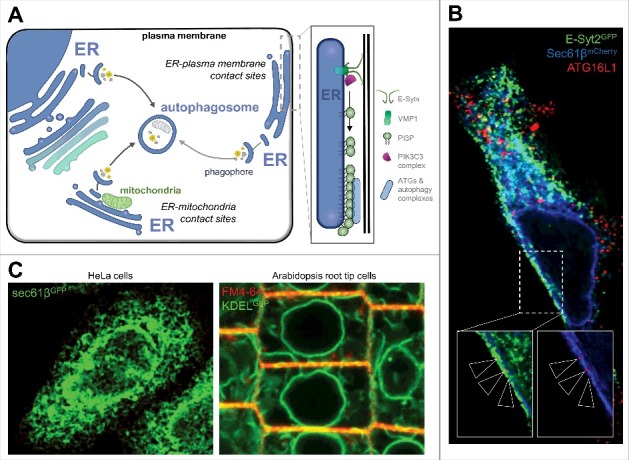
(A) Schematic overview of ER-driven contact sites implication in autophagy regulation. ER-mitochondria and ER-plasma membrane contact sites are shown. Details about ER-plasma membrane contribution to autophagosome biogenesis are shown on the right panel: extended synaptotagmins (E-Syts) dialog with ER-protein VMP1 promotes class III PI3K complex (PIK3C3) recruitment and allows phosphatidylinositol-3-phosphate (PI3P) local synthesis. PI3P positive domains of the ER membrane are in turn engaged in recruitments of several ATGs and non-ATGs proteins necessary for pre-autophagosome membrane assembly and phagophore biogenesis. (B) Illustration of autophagosome biogenesis at ER-plasma membrane contact sites. HeLa cells transfected for Sec61βmCherry (an ER marker, blue channel) and E-Syt2GFP (an ER-plasma membrane contact sites marker, green channel) were starved for 15 min, fixed, stained with anti-ATG16L1 (red channel) antibody and processed for confocal imaging. Cropped pictures (inserts) show ATG16L1 - considered as a marker of early stages of autophagosome biogenesis - structures at the interface of ER membrane (in blue) and ER-PM contact sites (in green), illustrating the local appearance of pre-autophagosome at the ER membrane facing the plasma membrane, in E-Syts positive domains. (C) Illustration of ER network distribution in mammals' cells (HeLa cells, Sec61βGFP as ER marker, in green) and plant cells (Arabidopsis root tip cells, KDELGFP as ER marker, in green and FM4-64 as plasma membrane dye, in red).

Intracellular communication and exchanges between organelles has long been thought to depend on vesicular transport and diffusion of molecules in the cytosol. However growing evidence push membrane contact sites in the front of stage of organelle dialogs and signaling regulation. The Endoplasmic Reticulum (ER), which is the most extended intracellular membrane system, has contact sites with most of the cell organelles including the plasma membrane (PM).^1^ Macro-autophagy (hereafter referred to as autophagy), is a major lysosomal degradative pathway for intracellular components conserved in eukaryotic cells. Autophagy which is stimulated upon stress induction, requires ER membrane for the biogenesis of autophagosome, the main organelle of autophagy, which will later fuse with lysosome.^2^ Autophagy and autophagosome biogenesis are initiated by the recruitment of evolutionary conserved ATG (autophagy-related, such as ATG1, ATG5, ATG8, ATG12, ATG16L1, etc.) proteins and non-ATG proteins (such as Vps34, Vps15, AMBRA-1) engaged at the ER membrane in a region named omegasome. These regions are characterized by accumulation of PI3P^3^ and consequent recruitment of specific PI3P binding proteins such as DFCP1 and WIPIs. DFCP1labels at early stage the ER cradle known as omegasome and WIPI2, yeast orthologue of ATG18, is recruited to the phagophore,^4^ a cup shaped structure that ultimately closes-up to form the double membrane bound autophagosome. The ER-mitochondria contact sites have been shown to be a place for the recruitment of the ATG machinery and autophagosome assembly.^5^ However, our recent work shows that ER-PM is another site for the recruitment of early actors in autophagosome biogenesis.^6^ Indeed, we show that the extended synaptotagmins (E-Syts) proteins, key-regulators of ER-PM tethering,^7-9^ are directly involved in autophagosome biogenesis. More particularly, we show that E-Syts are engaged, after nutritional stress sensing, in the local targeting of PI3P synthetizing complex via VMP1 (vacuole membrane protein 1) mobilization, a step known to be responsible for autophagosome biogenesis initiation (Fig. 1A and B). Interestingly, the VMP1 protein, originally identified as a regulator of PI3P synthesis during autophagosome biogenesis initiation^10-12^ was recently reported to be physically associated with ER-driven contact sites at mitochondria, endosomes and lipid droplets,^13^ making sense with our own observations showing the targeting of VMP1 at ER-PM contact sites (ER-PMcs) engaged in autophagosome biogenesis.^6^ Importantly, it has recently been shown that VMP1 controls the formation of these ER contacts by controlling SERCA (Sarco/Endoplasmic Reticulum Calcium ATPase) activity.^14^ Loss of VMP1 greatly increases these contact sites and causes stable association of isolation membranes with the ER. This in turn affects autophagy, impeding the dissociation of ATG proteins from isolation membranes upon closure, thus blocking autophagosome formation. Notably, this dissociation process appears to require clearance of PI3P,^15^ thus suggesting a role for VMP1 and contact sites in the fine-tuning of the key phospholipid in autophagosome biogenesis.

### Plants and mammalian ER-driven contact sites as models: Open questions

Cytoplasmic spreading and “exploring” by ER are common feature across eukaryotic cells. The importance of ER engagement in membrane contact sites stems out from the evolutionary conserved capability of the ER to promote tethers with other endomembranes, from yeast to plants and mammals.^16^ Some functions of ER-PMcs have been identified and are conserved among metazoan, as for example in Ca^2+^ signaling,^17^ some others have been evoked more recently and so far now ascertained only in few models, like the ER-PMcs functioning in autophagosome biogenesis in mammalian cells.^6^ However, even if in plants cells ER-PMcs were not formally identified as crucial platforms for autophagosome biogenesis, the presence of pre-autophagosomal markers on ER membrane at the immediate vicinity of PM is in favor of a similar situation.^18^ In the light of our recent results, it would be definitely worth deeply searching for a potential role of plant ER-PMcs in autophagy.

One way to unveil the different conserved functions of the ER-PMcs is to analyze the different proteins that have been found localized in there, mostly categorized as ER-proteins. For example the plant PM proteins STIM1 and STIMATE, regulating Ca^2+^ signaling, have been found in mammalian^18^ cells. Ist2, regulating ion transport, seems to be specific to yeast; NET proteins, binding actin cytoskeleton, seem to be specific to plants.^19^ On the other hand, the family of sterols binding proteins known as ORP/Osh, were found in animal, yeast and plants, thus suggesting that the role of ER-PMcs in lipid homeostasis is highly conserved. However, while in yeast ORP/Osh proteins where found to regulate PI4P metabolism^20-22^ and sterol and PS transport,^23^ a clear cut experimental evidence of a lipid transport occurring at plant ER-PMcs is still missing, thus a role of plants ER-PMcs in lipid homeostasis is still an open question. Among the ER resident proteins involved in contacts with PM found in yeast, animals and higher plants, are the E-Syts (Tricalbin1/2/3 in yeast) and VAP (Scs2/22 in yeast) proteins. E-Syts^8^ belong to the family of proteins known as Synaptotagmins (Syts), originally called like this because particularly abundant in synaptic vesicle and secretory granule in neurons and endocrine cells, where they play a key role in Ca^2+^ induced exocytosis.^24^ The major functional difference between Syts and E-Syts (for extended synaptotagmin) is that while Syts can translocate along the secretory pathway and mediate fusion of secretory vesicles, E-Syts are ER resident membrane proteins that bridge ER and PM for lipid exchange without involving membrane fusion.^25^ Syt1 has 2 C2 domains that bind calcium and lipids. E-Syts contain additionally C2 domains (3 to 5) and a conserved Synaptotagmin-like mitochondria lipid binding protein (SMP) domain, shared among proteins of the tubular lipid-binding protein (TULIP) superfamily localized at multiple contact sites.^26^ The Arabidopsis Synaptotagmin 1 (AtSyt1) resembles to both Syt and E-Syt and also localizes and plays roles at ER-PMcs.^27^ The second type of ER -resident proteins found in ER-PMcs are VAP proteins, where VAP stands for VAMP-associated proteins. They are highly conserved integral ER membrane proteins involved in varied cellular functions, including lipid transport and homeostasis, membrane trafficking and neurotransmitter release. Plants VAP, VAP27 also localize to ER-PMcs, however differently from metazoans, the two proteins (Syt1 and VAP27) label two different ER subdomains, suggesting that different type of ER-PMcs exist, at least in plants.^26^ So do they are different ER-PMcs with different functions? Do ER-PMcs play different roles in different organisms?

In plant cells the ER network appears very different from animal ones (Fig. 1C), mainly because a huge vacuole squeezes the cytoplasm close to the cell cortex obliging a great proximity between organelles, so for instance both Golgi bodies and ER appear very close to the PM.^28^ Special structures, found only in plant PM, are plasmodesmata (PD), a kind of pore mainly used as cell-cell communication structures.^29^ SYT1 and VAP27 are also found here and they both interact with plasmodesmata resident reticulon.^30^ Furthermore, they both interact with viral proteins and mediate trafficking of viral replications complex.^31^^,^^32^ Such structures represent specialized plant ER-PMcs, in which both ER actin and PM converge creating a trafficking route toward which macromolecules as well as virus particles move trough. Considering that, one may ask whether also in animals ER-PMcs could play roles in viral response. Interestingly enough functions of ER-Mitochondria contacts sites (ER-MTcs) in viral response have been described.^33^ Furthermore, in animal cells autophagy is known to play complex roles in virus replication.^34^ Thus, considering the intricate roles of autophagy in viral infection and the link between both ER-PMcs and ER-MTcs with autophagy, as already mentioned, one may wonder if the relationship between autophagy and viral infection passes by ER contact sites, at mitochondria and/or plasma membrane.

Finally, some other key questions would be worth to assess are: are the autophagosomes produced at ER-MTcs vs ER-PMcs different? Are they engulfing different cargos? Is the engagement of different sets of ER-driven contact sites in the biogenesis of autophagosome a possibility for the cell, in plants and in mammals, to adapt to different types of stresses?

Interestingly, a role for the ER stress sensor PERK in the regulation of ER-PM appositions, through the modulation of the actin cytoskeleton, has been recently discovered. However, whether this mechanism correlates with the autophagy pathway was not explored.^35^ Can ER-PMcs and ER-MTcs, beyond the recruitment of the autophagy machinery, be specialized domains for lipid transfer and signaling to the autophagy pathway? What are the functions of ER-other organelles contact sites? Should VMP1 protein be considered as a molecular linker between contact sites and autophagosome biogenesis machineries? In this context, it is interesting to note that different Osh/ORP proteins have been found localizing to different organelles. The meaning of these ORP-VAP interactions at ER-organelles contacts is the lipid transfer between organelle membranes,^36^ possibly for signaling pathways activation, but probably also to control membrane compositions. Indeed, ORP1L localizes to late autophagosomes and it has been proposed to regulate transport and positioning of late autophagosomes upon cholesterol and—under low-cholesterol conditions—contacts the ER protein VAP-A, forming thus ER-autophagosome contact sites.^37^ Whether other ER-driven contact sites are engaged in autophagy to initiate and/or terminate the process are open questions in an emerging and fascinating aspect of ER membrane dynamics during stress-response(s).
